# Anticancer effects of zinc ion-mediated DNA demethylation in oesophageal squamous cell carcinoma

**DOI:** 10.3389/fphar.2025.1559675

**Published:** 2025-05-27

**Authors:** Bin Zhou, Changchun Wang, Yueyu Huang, Xuping Yang, Ting Ye, Lize Shen, Qiaoli Lv, Weimin Mao, An Zhao

**Affiliations:** ^1^ Department of Surgery, The First Affiliated Hospital of Soochow University, Suzhou, China; ^2^ Department of Surgery, Taixing People’s Hospital, Yangzhou University, Taizhou, China; ^3^ Department of Thoracic Surgery, Zhejiang Cancer Hospital, Institute of Cancer and Basic Medicine (ICBM), Chinese Academy of Sciences, Hangzhou, China; ^4^ Zhejiang Cancer Institute, Zhejiang Cancer Hospital, Hangzhou Institute of Medicine, Chinese Academy of Sciences, Hangzhou, Zhejiang, China; ^5^ Postgraduate training base Alliance of Wenzhou Medical University (Zhejiang Cancer Hospital), Hangzhou, Zhejiang, China; ^6^ Department of Clinical Laboratory, Taixing People’s Hospital, Yangzhou University, Taizhou, China; ^7^ LC-Bio Technologies, Hangzhou, Zhejiang, China; ^8^ Jiangxi Key Laboratory of Tumour Metastasis of Jiangxi Health Commission, Jiangxi Cancer Hospital, Nanchang, Jiangxi, China

**Keywords:** oesophageal squamous cell carcinoma, zinc ions, DNA methylation, gene expression, metallothioneins, combination drug therapy, biomarkers

## Abstract

**Background:**

Abnormalities in trace elements and the incidence of oesophageal squamous cell carcinoma (ESCC) have been reported in China. Zinc ions (Zn^2+^) are known to regulate DNA methylation by stabilizing methylase activity. However, the relationship between DNA methylation and Zn^2+^ dysregulation in ESCC cells remains unclear. In this study, we examined changes in the biological behavior of ESCC cells treated with or without Zn^2+^.

**Methods:**

Biological behaviour changes in ESCC cells treated with or without Zn^2+^ were analysed. Differences in the methylome and transcriptome of Zn^2+^-treated cells were determined by reduced representation bisulfite sequencing and RNA sequencing. An MTT cell viability assay was used to evaluate the cytotoxicity of cisplatin combined with Zn^2+^.

**Results:**

Zn^2+^ can inhibit the malignant biological behaviour of ESCC cells. CpG methylation levels of promoter regions were decreased after Zn^2+^ treatment in both ESCC and control cells. The degree of DNA methylation of genes encoding the metal ion-binding factors MT1E, MT1H and MT1X was significantly decreased, but their RNA expression levels were significantly increased after Zn^2+^ treatment. Zn^2+^ may enhance the expression of metallothioneins (MTs) via positive feedback through methylation regulation mechanisms. *In vitro* assays showed that the IC50 of Zn^2+^ in ESCC cells was significantly lower than that in cells treated with cisplatin alone. In addition, ECa patients with high MT1E expression had a better prognosis.

**Conclusion:**

Zn^2+^ can reduce the methylation level and malignant biological behaviour of ESCC cells. The combination of Zn^2+^ and cisplatin increases ESCC inhibition. Further study of MTs as biomarkers and targets in ESCC is warranted.

## Introduction

Oesophageal cancer (ECa) is the eighth most common cancer worldwide, with two primary histological subtypes: oesophageal squamous cell carcinoma (ESCC) and oesophageal adenocarcinoma (EAC) (Sung et al., 2021). China exhibits a notably high incidence of ESCC, accounting for nearly half of all ECa-related deaths worldwide (Abnet et al., 2018; Wang et al., 2025). Although significant progress has been made in surgical and multimodal therapies, the 5-year overall survival rate remains at approximately 50% (Yang et al., 2021). One critical factor in ESCC progression is the enhanced invasive behaviour of tumor cells, which plays a major role in lymphatic metastasis. However, the molecular mechanisms driving ESCC development and aggressiveness are not fully understood (Lin et al., 2016; Chang et al., 2017). Moreover, ESCC is found at a high incidence in some areas of China, suggesting a potential interaction between related genes and the environment.

Zinc ions (Zn^2+^) are important trace elements necessary for the activity of ∼2000 transcription factors and more than 300 enzymes, with an important role in many physiological functions (Vallee and Falchuk, 1993; Levaot and Hershfinkel, 2018). Different organs depend on zinc in various ways, and an excess or deficiency can cause several diseases, such as dermatitis, neuropathy, metabolic bone disease, immune deficiency and cancer (Ho, 2004). Several DNA methylation-related epigenetic factors (DNMT1, DNMT3a and MeCP2) have a CXXC domain, which binds to Zn^2+^ and is required for catalytic activity (Yusuf et al., 2021), and intracellular Zn^2+^ deficiency can affect cell proliferation and the degree of DNA methylation (Chen et al., 2021; Yusuf et al., 2021). In addition, ERK-directed phosphatase PP2A activity is reduced when Zn^2+^ is added to cell lysates; thus, Zn^2+^ may influence DNMT1 via the MEK/ERK pathway (Huiliang et al., 2021; Zeng et al., 2025). Taken together, these findings suggest that there is a relationship between DNA methylation and intracellular Zn^2+^ abnormality-induced ESCC; however, no direct experimental data have been reported thus far. Therefore, we aimed to explore the relationship between DNA methylation and Zn^2+^ abnormalities in ESCC cells and its potential translational application value.

## Materials and methods

### Cell culture

Human normal oesophageal epithelial cells (HEECs) and KYSE-450 were obtained from the American Type Culture Collection (ATCC, United States). The ESCC cell (ECA109) was obtained from the cell bank at the Chinese Academy of Science, and the cells were authenticated by short tandem repeat. The cells were cultured in 1640 medium (Gibco, United States) supplemented with 10% FBS (Gibco, United States), 100 U/mL penicillin sodium and 100 μg/mL streptomycin sulfate (Gibco, United States) at 37°C in humidified air containing 5% CO_2_.

### Cell treatment with zinc ions

Cells were cultured in six-well plates at 5 × 10/well. After washing twice with PBS, the cell culture medium was replaced with RPMI 1640 medium (control group) or RPMI 1640 medium supplemented with 50 µM ZnSO4 (zinc ion-treated group) (Choi et al., 2018). After 48 h of culture, cells were harvested for flow cytometry or protein or nucleic acid extraction for subsequent experiments.

### Flow cytometric analysis and Western blot analysis of apoptosis

Annexin V-FITC and propidium iodide staining (PI) were used to evaluate the effects of zinc ions on apoptosis in ESCC and HEEC cells. After the cells were incubated with the different media for 48 h, they were collected, centrifuged, and incubated with Annexin V-FITC/PI for 15 min at room temperature in the dark, followed by BD FACSVia flow cytometry. The cells were lysed with a modified RIPA buffer in the presence of a protease inhibitor cocktail and total protein concentration was measured using the BCA assay. Next, 20 µg of protein was separated via 15% SDS - PAGE using pre-cast gels and electro-transferred to 0.22 µm PVDF membranes. The membranes were blocked with 5% skim milk in TBST (Tris-buffered solution containing 0.1% Tween - 20) for 2 h at room temperature. Subsequently, the membranes were incubated with primary antibodies, namely, Anti - Cleaved PARP1 antibody ([E51], ab32064, Abcam) and BAX Polyclonal Antibody (50599-2-Ig, Proteintech), overnight at 4°C, with dilutions as per the manufacturers’ instructions. GAPDH served as the internal control. After washed with PBST three times, the membranes were incubated with HRP-conjugated secondary antibodies (Proteintech, 1:5,000) for 1 h at room temperature. Protein bands were visualized and analyzed using an enhanced chemiluminescence (ECL) detection system.

### DNA extraction and reduced representation bisulfite sequencing (RRBS)

Total DNA was extracted using a QIAamp Fast DNA Tissue Kit (Qiagen, Dusseldorf, Germany) following the manufacturer’s procedure. A260/280 ratios were assessed by spectrophotometry to determine the quality of the extracted DNA. The fragmented DNA samples were subjected to bisulfite conversion by using MspI (NEB, United States), and Accel-NGS Methyl-Seq DNA Library Kit (Swift, MI, United States) was utilized to attach adapters to single-stranded DNA fragments. The final library DNA was quantified using a Qubit fluorometer and a Quant-iT dsDNA HS Kit (ThermoFisher, Q32854). Bead-based SPRI clean-up was used to remove both oligonucleotides and small fragments and to change the buffer composition. Pair-end 2 × 150 bp (PE150) sequencing was performed using an Illumina NovaSeq™ 6000 (LC-Bio Technology Co., Ltd., Hangzhou, China). The methylation calls were filtered based on the minimum coverage >1.

### RNA extraction and RNA sequencing

Total RNA was isolated and purified using TRIzol reagent (Invitrogen, United States) following the manufacturer’s procedure. The amount and purity of each RNA sample were quantified using a NanoDrop ND-1000 (NanoDrop, United States). RNA integrity was determined using a 2100 Bioanalyzer system. Library preparation was performed by enrichment of poly-A tailed transcripts from total RNA, and the library type was strand-specific. The average insert size for the final cDNA library was 300 ± 50 bp. Pair-end sequencing (PE150, 2 × 150 bp) was performed using an Illumina NovaSeq™ 6000 (LC-Bio Technology Co., Ltd., Hangzhou, China).

### RT-qPCR

Total RNA was extracted using the FastPure Cell/Tissue Total RNA Isolation Kit V2 (Vazyme, Cat. RC112-01) according to the manufacturer’s instructions. cDNA was synthesized using the PrimeScript™ RT Master Mix (Takara, RR036A). Quantitative real-time PCR (RT-qPCR) was performed on a QuantStudio 5 real-time PCR system using the BrightCycle Blue Universal SYBR Green qPCR Mix with UDG (ABclonal, RK21220). The amplification reaction procedure was as follows: 95°C for 10 min, followed by 40 cycles of 95°C for 15 s and 60°C for 1 min. GAPDH was used as internal control for mRNA, and relative mRNA expression levels were calculated using the 2^^−ΔΔCT^ method. The primer sequences were as follows: MT1E forward: 5′-AAA​GGG​GCA​TCG​GAG​AAG​TG-3′, reverse: 5′-CCA​GGT​TGT​GCA​GGT​TGT​TC-3′; MT1H forward: 5′-CAT​CTG​CAA​AGG​GGC​GTC​AG-3′, reverse: 5′-CCT​GGA​TTT​TAC​GTG​TCA​TTC​TGT-3′; MT1X forward: 5′-CAC​GCT​TTT​CAT​CTG​TCC​CG-3′, reverse: 5′-GGT​CCA​TTT​CGA​GGC​AAG​GA-3′; GAPDH forward: 5′-CTG​AGT​ACG​TCG​TGG​AGT​CC-3′, reverse: 5′-GAG​GCA​TTG​CTG​ATG​ATC​TTG​A-3′.

### Bioinformatic analysis

Cutadapt (Martin, 2011) was used to remove reads that contained adapter contamination, low-quality bases and undetermined bases. Then, sequence quality was verified using FastQC (http://www.bioinformatics.babraham.ac.uk/projects/fastqc/). Reads that passed quality control were mapped to the *Homo sapiens* reference genome (GRCh38) using Bismark and Samtool (Li et al., 2009; Krueger and Andrews, 2011). For each cytosine site (or guanine corresponding to a cytosine on the opposite strand) in the reference genome sequence, the DNA methylation level was determined by the ratio of the number of reads supporting C (methylated) to that of total reads (methylated and unmethylated) using in-house scripts and MethPipe (Song et al., 2013). Differentially methylated regions (DMRs) were calculated by the R package MethylKit with default parameters (1,000 bp slide windows, 500 bp overlap, p value < 0.05) (Akalin et al., 2012).

The sequence quality of the IP and input samples was verified using fastp software (Chen et al., 2018) and HISAT2 to map reads to the *H. sapiens* reference genome (GRCh38) (Kim et al., 2015). Mapped reads were assembled by StringTie with default parameters, StringTiewas was used to determine the expression level of all mRNAs from the input libraries by calculating FPKM (total exon fragments/mapped reads (millions) × exon length (kB) (Pertea et al., 2015). Differentially expressed mRNAs were selected with log2 (fold change) > 1 or log2 (fold change) <−1 and p value <0.05 by the R package edgeR (Robinson et al., 2010).

### Transwell migration and invasion assay

For the migration assay, cells (1 × 10^5^) were plated in the upper chamber of a Transwell plate and cultured in serum-free medium with or without ZnSO_4_ for 24 h. Medium containing 10% FBS (500 µl) was added to the lower chamber to create a chemotactic gradient. Then the cells were fixed with 4% paraformaldehyde and stained with crystal violet (Beyotime). For the invasion assay, diluted Matrigel (Corning) (30 µl) was placed in the upper chamber of the Transwell plate to simulate the extracellular matrix and analyse cell invasion. five fields per membrane were selected for imaging (one central field and four peripheral fields). Cell counting was performed manually to ensure accuracy.

### Wound-healing assay

ESCC cells were cultured in six-well plates at 5 × 10^5^/well. When the cell monolayer reached 90% confluence, a uniform scratch was made with a sterile 200 μl pipette tip to create a wound gap. Cells were then washed twice with PBS to remove excess cells. Cells were treated with 50 µM ZnSO_4_ to be compared to the control condition. Images of the cell monolayer were taken under a microscope at 0, 12, 24, and 48 h. Wound-healing efficiency was assessed by quantifying the scratch area using ImageJ.

### MTT cytotoxicity assay

We used an MTT assay to evaluate the cytotoxic effects of cisplatin in combination with Zn^2+^. Briefly, 2 × 10^4^cells were seeded per well in a 96-well plate and cultured for 24 h. One group of wells received serial concentrations of cisplatin (0.01, 0.1, 1, 10, and 100 µM), whereas another group was treated with 50 µM ZnSO_4_plus various concentrations of cisplatin (0, 0.1, 0.5, 1, 1.5, 2, and 2.5 mM). After 48 h of incubation, the medium was replaced with fresh medium containing 2 mg/mL MTT, and plates were incubated for an additional 4 h at 37°C. The absorbance of each well was assessed at 570 nm using an ELISA plate reader (Bio-Tek Instruments) with a reference wavelength of 630 nm.

### Statistical analysis

Each condition (control and Zn^2+^-treated) was performed in six replicates per experiment, and all experiments were repeated three times. All statistical analyses were conducted using SPSS 20.0 statistical software and GraphPad Prism 5.0 software. Concentration differences were analysed by using the Mann–Whitney U, Friedman or Wilcoxon test. The significance of the multiple comparisons was considered statistically significant a P value less than 0.05.

## Results

### Zn^2+^ inhibits the malignant biological behaviour of ESCC cells

**FIGURE 1:**
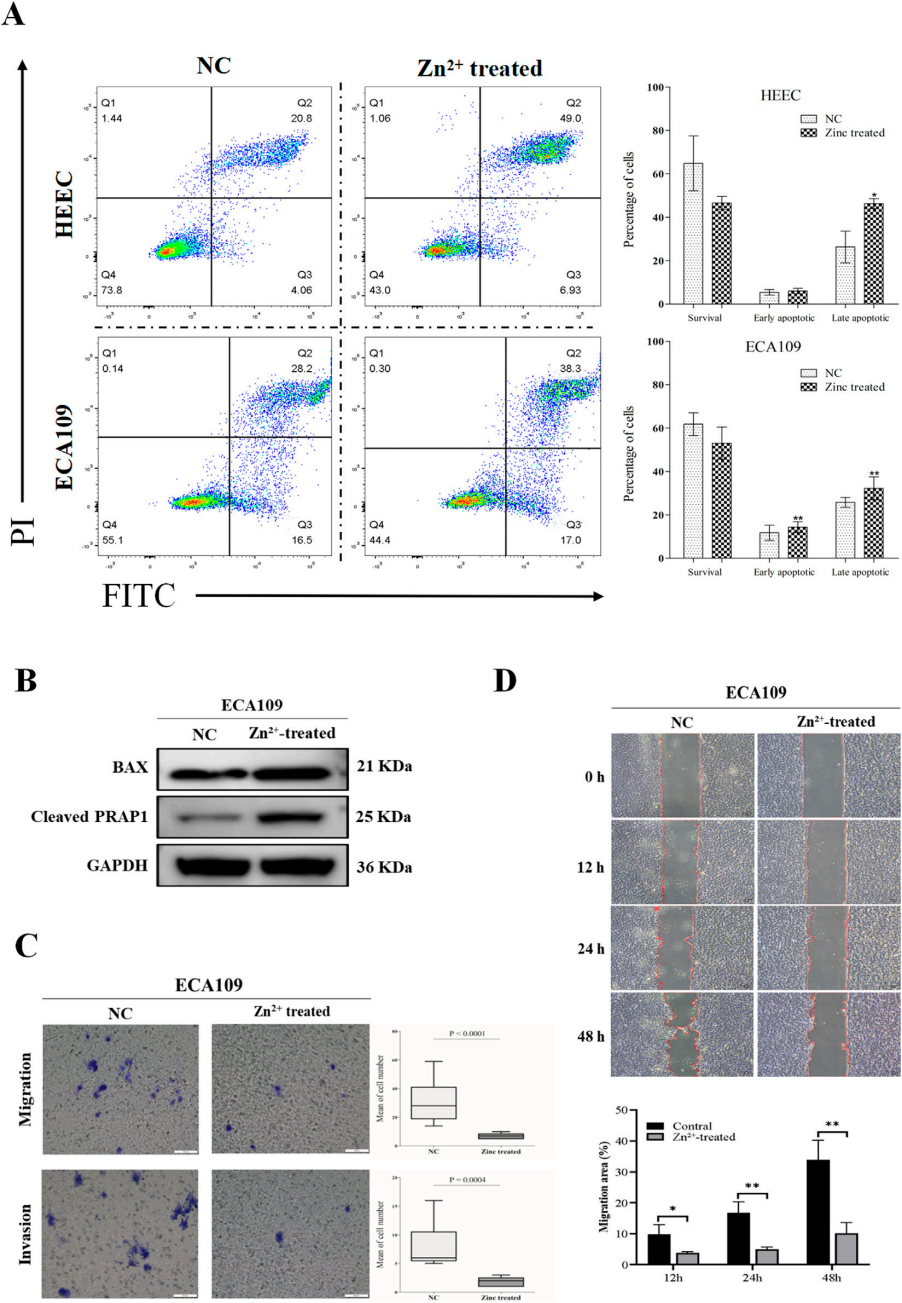
Zn^2+^ inhibits the malignant biological behaviour of ESCC cells. **(A)** Apoptosis of both ECA109 and HEEC cells was determined by flow cytometry-based Annexin VFITC/PI apoptosis assay 48 h after treated with or without Zn^2+^. Histograms show the percentage of survival and apoptotic cell number. **(B)** Western blot analysis indicating the expression and activation of BAX and Cleaved PRAP1 in ECA109 cells with Zn^2+^ treatment. GAPDH was used as a protein loading control. **(C)** Transwell assay of ECA109 cell lines after Zn^2+^ treatment. The scale bar is 100 μm. Histograms show the percentage of migrated and invasive ECA109 cell number. **(D)** (Top) Representative microscopy images of wound closure in Zn^2+^-treated ESCC cells at 0, 12, 24, and 48 h (Bottom) Quantitative analysis of the scratch wound healing assay. Data are presented as mean ± SD. *p < 0.05; **p < 0.01.

After 48 h of Zn^2+^ treatment, we analyzed apoptosis in both ESCC and HEEC cells using flow cytometry. As shown in Figure 1A, Zn^2+^ treatment significantly increased apoptosis in both cell types when compared to the untreated controls. Notably, the proportion of early apoptotic cells was significantly higher in ESCC than in HEEC cells. Meanwhile, Western blot analysis revealed an increase in the expression levels of BAX and cleaved PARP1 in Zn^2+^-treated ESCC cells compared to the control group (Figure 1B). Given that BAX is a key pro-apoptotic protein involved in mitochondrial outer membrane permeabilization (MOMP) and that cleaved PARP1 is a hallmark of caspase-mediated apoptosis (Tang et al., 2019), these results suggest that Zn^2+^ primarily induces apoptosis through the intrinsic (mitochondrial) pathway.

We next examined the effect of Zn^2+^ on the migration and invasion of ESCC cells and found Zn^2+^ to decrease the cellular motility of ESCC cells. At 24 h, Zn^2+^ inhibited more cells from crossing the Transwell membrane (Figure 1C). In a Transwell invasion assay, cells treated with Zn^2+^ also demonstrated inhibited invasion compared with control cells (Figure 1C). Additionally, a wound healing assay performed at 0, 12, 24, and 48 h revealed that Zn^2+^ significantly impaired ESCC cell migration over time (Figure 1D). These results indicate that Zn^2+^ directly inhibits ESCC cell migration and invasion, in addition to its pro-apoptotic effects.

### Differences in the methylome of Zn^2+^-treated cells by RRBS analysis

**FIGURE 2:**
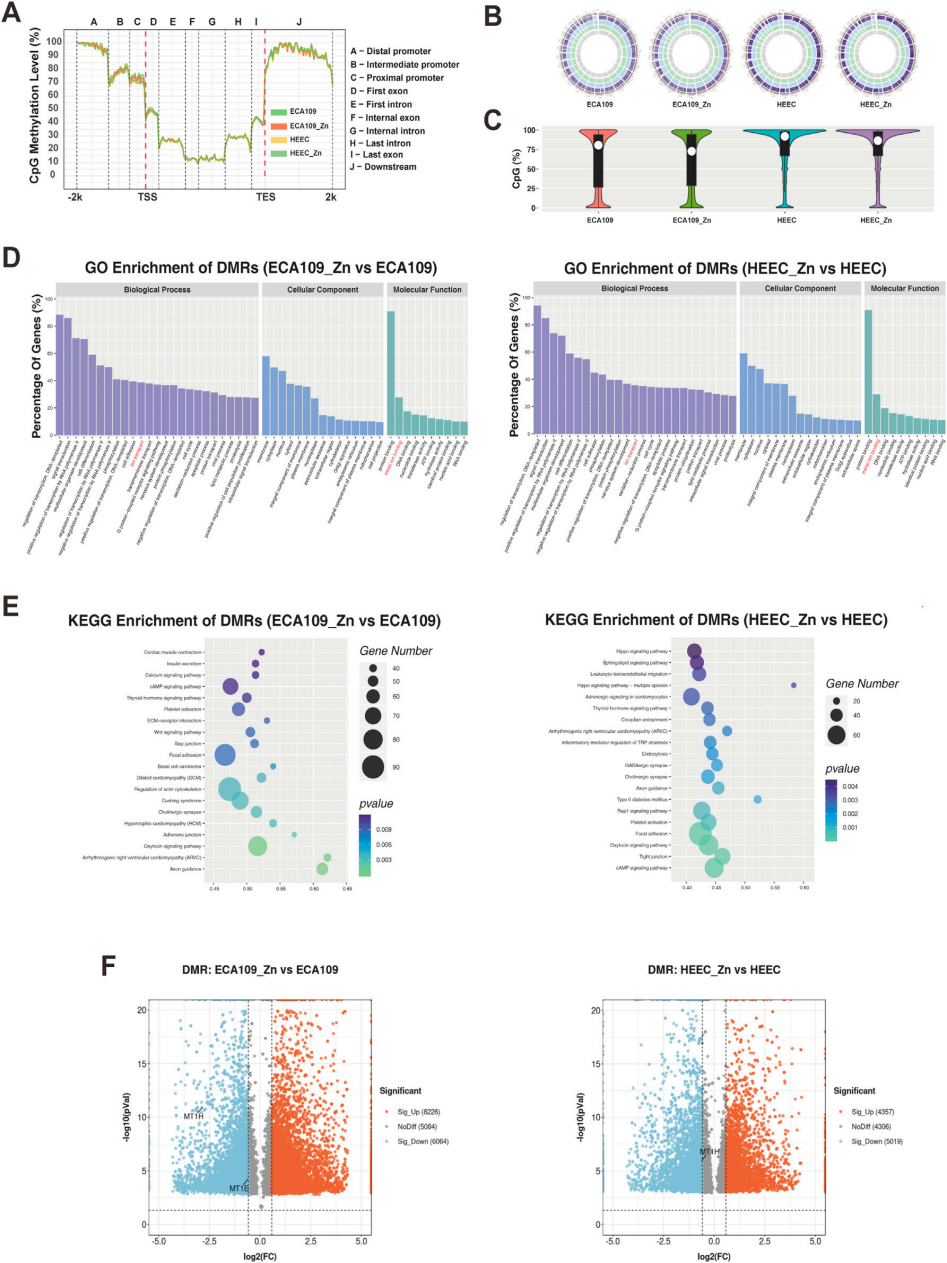
Differences in the methylome of Zn^2+^-treated cells by RRBS analysis. **(A)** Line graph of CpG methylation levels of different gene body regions in ECA109 and HEEC cells after treatment with or without Zn^2+^. **(B)** Circos plots of the overall CpG methylation levels distributed on chromosomes. **(C)** Violin plots of the CpG methylation levels of all promoter regions. **(D, E)** GO and KEGG enrichment analysis of differentially methylated gene regions (DMRs) in ECA109_Zn verse ECA109 and HEEC_Zn verse HEEC. **(F)** Volcano plots of DMRs included upregulated and downregulated regions in ECA109_Zn verse ECA109 and HEEC_Zn verse HEEC.

RRBS was performed after cells were treated with or without Zn^2+^. All four samples showed hypermethylation in the promoter regions, with the methylation level of the distal promoter region close to 90% and that of the intermediate and proximal promoter regions over 70% (Figure 2A). The overall CpG methylation levels of the four samples were uniformly distributed on chromosomes, with a high degree of methylation (circos plot chrobin size = 500 k bp) (Figure 2B). A violin plot was generated to display average CpG methylation in the distal, intermediate and proximal promoter regions of the samples; the overall CpG methylation level of promoter regions was decreased after Zn^2+^ treatment in both HEEC and ESCC cells (Figure 2C). These results indicate that Zn^2+^ may affect DNA methylation.

Then, GO and KEGG enrichment analyses were performed for DMRs (promoter bin region = 2,000 bp). The biological process category showed enrichment in the regulation of transcription, signal transduction, cell adhesion, ion transport and the cell cycle; associations with protein binding, metal binding and DNA binding were found for the molecular function category (Figure 2D). Interestingly, ion transport and metal ion binding were observed for both HECC and ESCC cells, suggesting that Zn^2+^ may play a role in the regulation of methylation of related genes. KEGG pathway analysis revealed that DMRs were highly associated with pathways in ESCC and HEEC cells that were different (Figure 2E). All DMRs are plotted as volcano plots in Figure 2F. A total of 4,357 DMRs were upregulated and 5,019 DMRs downregulated in HEEC cells treated with Zn^2+^; 8,226 DMRs were upregulated and 6,064 DMRs downregulated in ESCC cells treated with Zn^2+^. Notably, the degree of DNA methylation of the metal ion-binding factor genes MT1E and MT1H was significantly decreased.

### Differences in the transcriptome of Zn^2+^-treated cells by RNA-seq analysis

**FIGURE 3:**
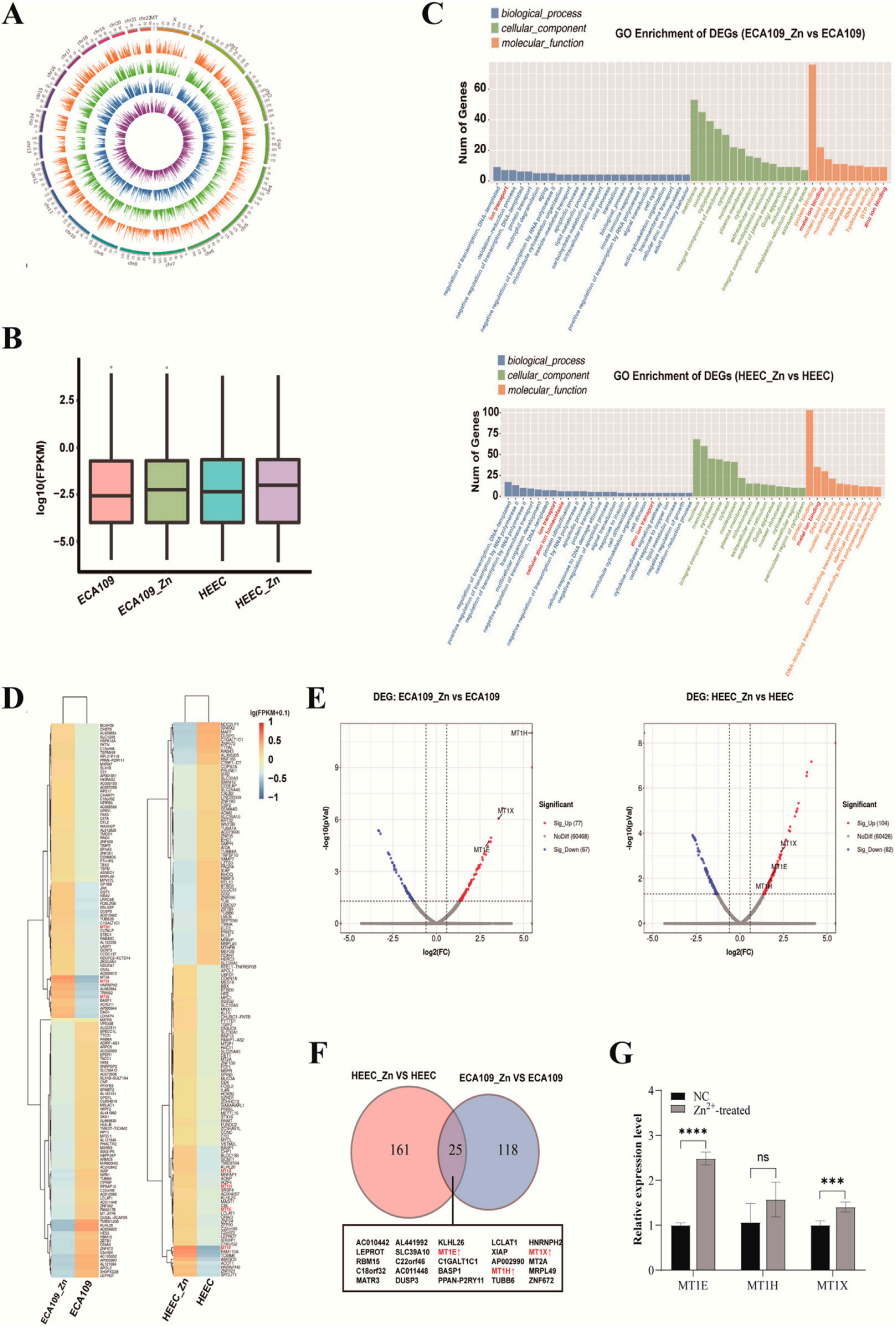
Differences in the transcriptome of Zn^2+^-treated cells by RNA-seq analysis. **(A)** Circos plot of the expressed genes distributed on chromosomes in ECA109 and HEEC cells with or without Zn^2+^ treatment. **(B)** Box plot of the overall gene expression levels in ECA109 and HEEC cells with or without Zn^2+^ treatment. **(C)** GO enrichment analysis of differentially expressed genes in ECA109_Zn verse ECA109 and HEEC_Zn verse HEEC. **(D)** The heatmap of top 100 DEGs found by GO enrichment analysis in ECA109_Zn verse ECA109 and HEEC_Zn verse HEEC. **(E)** Volcano plots of DEGs included upregulated and downregulated regions in ECA109_Zn verse ECA109 and HEEC_Zn verse HEEC. **(F)** Venn plot of shared genes in DEGs between ECA109_Zn verse ECA109 and HEEC_Zn verse HEEC. **(G)** RT-qPCR validated the expression level of MT1E, MT1H and MT1X in cells. Data are presented as mean ± SD from three independent experiments. ns, not significant; ***p < 0.001; ****p < 0.0001.

Zn^2+^-treated cells were also analysed by RNA-seq. Overall, the expressed genes of four different samples are distributed on all chromosomes (Figure 3A). After Zn^2+^ treatment, the average level of overall gene expression was slightly increased in both ESCC and HEEC cell lines (Figure 3B), suggesting that these upregulated genes may be associated with decreased methylation.

Differential expression analysis between the cell lines was next performed using the chi-square test (FDR ≤ 0.05 and fold change ≥ 2). There were 186 differentially expressed genes (DEGs) in the HECC cell line and 143 in the ESCC cell line (Figure 3F). The results of GO analysis revealed upregulated DEGs to be significantly enriched in the biological processes of ion binding and transport (Figure 3C). The top DEGs in the two groups are presented in heatmap and volcano plots, and expression of Zn^2+^ transporter-related genes, such as MT1E, MT1H and MT1X, was significantly increased after treatment with Zn^2+^ (Figures 3D, E). In addition, the intersection of DEGs between the two groups showed 25 common DEGs, including MT1E, MT1H and MT1X (Figure 3F). To further validate the expression of the DEGs, we performed RT-qPCR analysis. The results showed that MT1E (P < 0.0001), MT1H (P = 0.0824), and MT1X (P = 0.0003) expression levels were increased in Zn^2+^-treated cells compared to the control group (Figure 3G).

### Integration of methylome and transcriptome data

**FIGURE 4:**
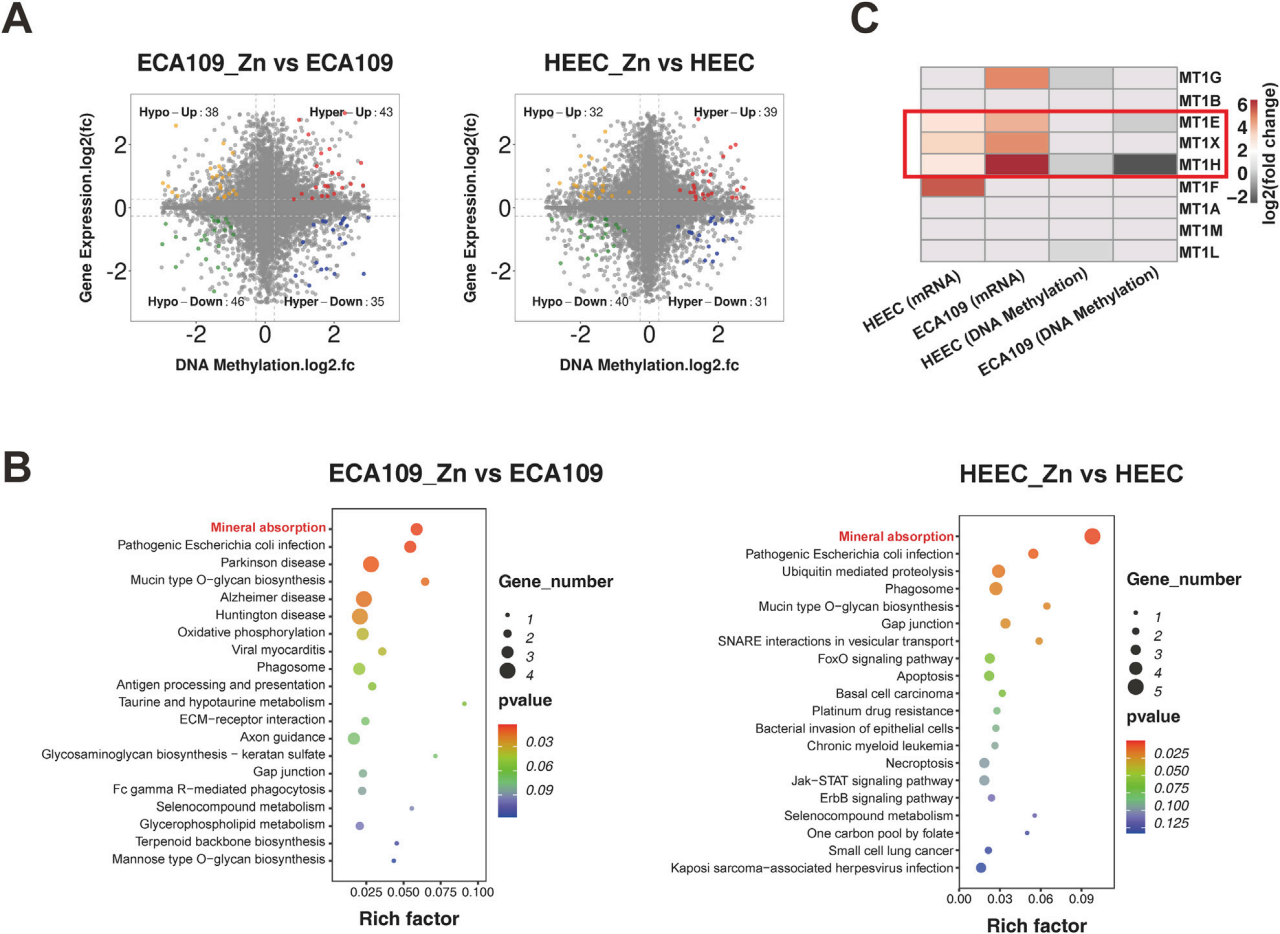
Integration of methylome and transcriptome data. **(A)** Starburst plots of the relationship between gene expression and DNA methylation in ECA109_Zn verse ECA109 and HEEC_Zn verse HEEC. **(B)** Dot plot of KEGG pathway enrichment analysis of concurrent DEGs found by conjoint analysis. **(C)** The heatmap of fold change of metallothionein subsets genes expression and DNA methylation levels in ECA109 and HEEC cells after the treatment of ZnSO4.

We next examined the association between gene expression and DNA methylation. Starburst plots in Figure 4A display DEGs with significant DNA methylation changes (hypermethylation or hypomethylation), and these DEGs were significantly enriched in the mineral absorption pathway in both ESCC and HEEC cells, as shown in Figure 4B. Furthermore, we focused on the metallothionein genes and found that DNA methylation levels of MT1E, MT1H and MT1X were significantly decreased but that their expression levels were increased after Zn^2+^ treatment (Figure 4C). These findings suggest that Zn^2+^ can enhance expression of metallothioneins via positive feedback through a methylation regulation mechanism.

### Potential application of Zn^2+^ in the treatment of ESCC

**FIGURE 5:**
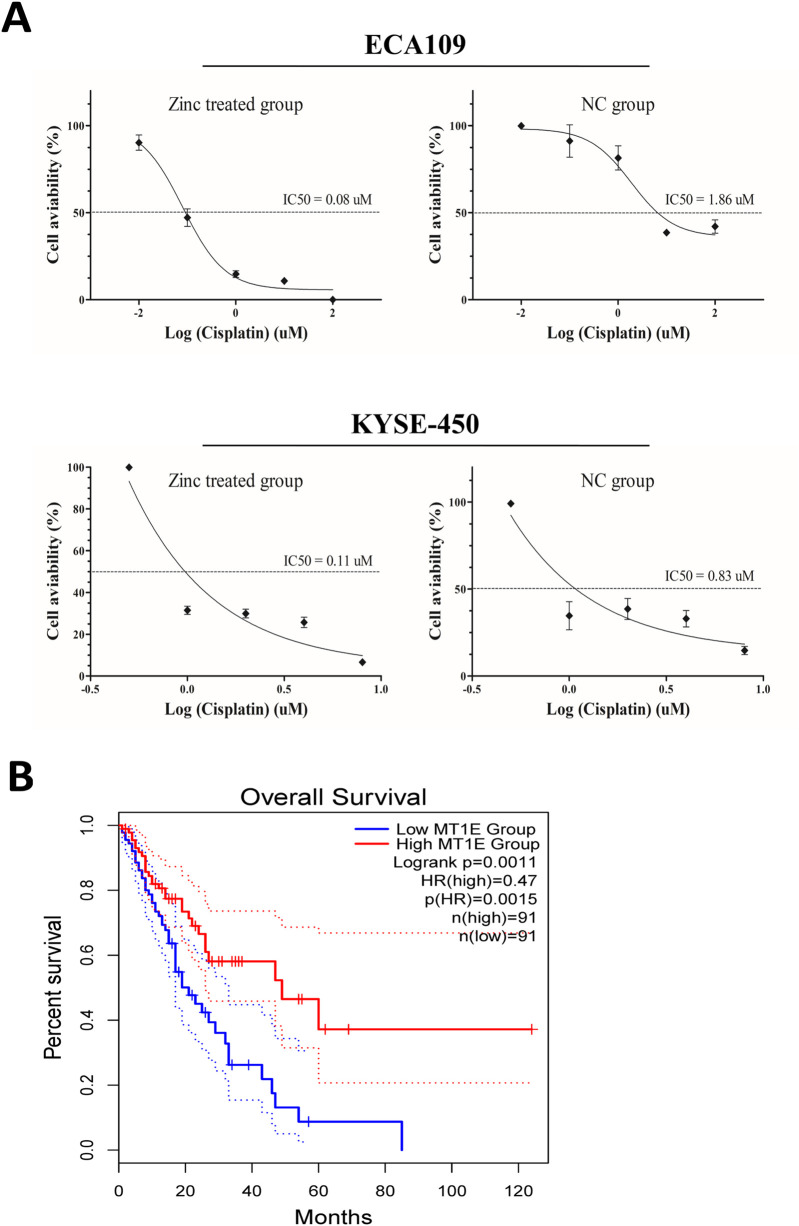
Potential application of Zn2+ in the treatment of ESCC. **(A)** Dose-response curve and half maximal inhibitory concentration (IC50) values of cisplatin with or without ZnSO4 treatment in ECA109 and KYSE-450 cell lines. **(B)** Kaplan-Meier analysis (overall survival) of ECa patients in the low and high MT1E expression groups via TCGA data analysis.

As cisplatin is the main chemotherapy drug for ESCC, we next investigated whether Zn^2+^ can be used in combination with cisplatin. *In vitro* assays showed that the IC50 of ESCC cells treated with Zn^2+^ was significantly lower than that of cells treated without zinc (Figure 5A). In addition, we used TCGA data to analyse MT1E, MT1H and MT1X expression levels in the assessment of ECa prognosis and found that ECa patients with high MT1E expression had a better prognosis (P < 0.01, Figure 5B). Although the detailed pathway changes induced by excess Zn^2+^ still need to be explored, Zn^2+^-MT binding status has potential value as a prognostic and therapeutic biomarker for ESCC.

## Discussion

Zn^2+^ is involved in various metabolic processes, including DNA synthesis and cell division (Levaot and Hershfinkel, 2018). Adequate zinc levels promote cell proliferation and tissue development, although different organs display distinct zinc requirements (Chen et al., 2022). In humans, the majority of Zn^2+^ intake is absorbed through the small intestine^2^ (Lee et al., 1989). The oesophagus and intestines are organs of the digestive tract, suggesting that the oesophagus may also have a natural ability to metabolize Zn^2+^. However, basic research on the role of Zn^2+^ and ECa development is lacking.

Zn^2+^ cannot freely enter cells and is transported passively via membrane transporters (Lichten and Cousins, 2009). Several studies have focused on the zinc-related transporter SLC30 or SLC39 (ZIP) family in cancer, reporting that abnormal Zn^2+^ in cancer cells caused by abnormal zinc transporters can affect AKT/PTEN and ERK/MAPK pathways (Zhu et al., 2021; Prasad et al., 2022). In addition to zinc transporters, four metallothionein isoforms (MT1, MT2, MT3 and MT4) can maximally bind seven zinc ions via tetrahedral coordination to cysteine residues, with a role for Zn^2+^ storage and releases (Krężel and Maret, 2021). Interestingly, we found that methylation of MT1E, MT1H and MT1X was significantly decreased and that expression of these genes was increased after ESCC cells were treated with Zn^2+^. When Zn^2+^ is increased in the environment, cells upregulate MT genes for the storage of Zn^2+^, and this Zn^2+^-MT regulatory pathway may be involved in disease and tumorigenesis.

Recent studies have reported an association between the expression of the MT family and tumor (Si and Lang, 2018; Merlos Rodrigo et al., 2020). MT1E is methylated in malignant melanoma and increases sensitivity to cisplatin-induced apoptosis (Faller et al., 2010). MT1H functions as a suppressor in hepatocellular carcinoma by regulating the Wnt/beta-catenin pathway (Zheng et al., 2017; Zhang et al., 2021). MT1X expression is downregulated in HCC tissues, and further research found that MT1X could inhibit the progression and metastasis of HCC, suggesting its use as a prognostic indicator (Liu et al., 2018). MT1 deletion in transgenic mice promotes the expression of IL-6, which leads to a significant reduction in angiogenesis, suggesting that MT1 may be involved in the angiogenesis process by regulating expression of proangiogenic factors (Giralt et al., 2002). Therefore, abnormal expression of MT genes may serve as a biomarker.

To explore the potential application of Zn^2+^ in ESCC treatment, we conducted *in vitro* experiments and found that Zn^2+^-treated ESCC cells exhibited a significantly lower IC50, suggesting that Zn^2+^ may enhance cisplatin sensitivity by modulating DNA methylation and gene expression involved in drug response. Moreover, numerous studies have demonstrated that cisplatin resistance is a complex process influenced by multiple factors beyond DNA methylation, including altered drug uptake and detoxification (Chen and Chang, 2019), enhanced DNA repair mechanisms (e.g., nucleotide excision repair and mismatch repair) (Kelland, 2007), other epigenetic modifications such as histone alterations and non-coding RNAs(Lumpp et al., 2024), activation of survival signaling pathways (e.g., NF-κB and Akt/p53) (Navaei et al., 2022), and metabolic reprogramming involving glucose, lipid, and amino acid metabolism (Pervushin et al., 2024). These mechanisms collectively drive cisplatin resistance in ESCC.

Furthermore, epidemiological studies have shown a link between dietary Zn^2+^ deficiency of ESCC (Abnet et al., 2005; Ma et al., 2018). Studies from Linxian, a high ESCC incidence area in China, have shown that serum Zn^2+^ concentrations are inversely associated with the risk of cancer development (Abnet et al., 2005). We analysed the epigenetic effects of Zn^2+^ on ESCC and HEEC cells *in vitro* for the first time and found that Zn^2+^ has the ability to alter ESCC epigenetic status and related downstream pathways. The regulation of MTs induced by Zn^2+^ deficiency may be related to the development and progression of ESCC. To the best of our knowledge, this is the first study to evaluate the potential combined treatment value of zinc ions and platinum in ESCC cells, suggesting that it is worth further evaluating the specific drug solubility of the two *in vivo* and *in vitro* experiments, including the combination index method (Chou, 2010). Future studies are needed to clarify the interaction between Zn^2+^ and MTs and their regulatory mechanisms, such as to investigate the effects of Zn^2+^ combined with cisplatin on tumor growth, metastasis, and epigenetic changes in xenograft mouse models or to study the Zn^2^-dependent drug sensitivity by CRISPR-cas9 knockdown or overexpression of Zn^2+^ transporter. And, to get more detail about Zn^2+^ driven effect as drug adjuvant in clinical application.

## Conclusion

In summary, Zn^2+^ reduces global DNA methylation and inhibits the malignant behavior of ESCC cells. Notably, combining Zn^2+^ with cisplatin further enhances the inhibitory effects on ESCC. We propose that metallothioneins warrant further investigation as potential biomarkers and therapeutic targets in ESCC.

## Data Availability

The RNA-seq and RRBS data generated in the present study have been deposited in the Genome Sequence Archive in BIG Data Center, Beijing Institute of Genomics, Chinese Academy of Sciences under accession number HRA011558 (publicly accessible at https://ngdc.cncb.ac.cn/gsa-human).
